# Efficacy comparison between long-term high-dose praziquantel and surgical therapy for cerebral sparganosis: A multicenter retrospective cohort study

**DOI:** 10.1371/journal.pntd.0006918

**Published:** 2018-10-22

**Authors:** Daojun Hong, Huiqun Xie, Hui Wan, Ning An, Chunhua Xu, Jun Zhang

**Affiliations:** 1 Department of Neurology, Peking University People’s Hospital, Beijing, China; 2 Department of Neurology, The first affiliated hospital of Nanchang University, Nanchang, China; 3 Clinical Department, Jiangxi provincial institution of parasitic diseases, Nanchang, China; 4 Department of Neurosurgery, The first affiliated hospital of Nanchang University, Nanchang, China; University of Kelaniya, SRI LANKA

## Abstract

**Background:**

Sparganosis is a parasitic infection caused by the plerocercoid larvae of *Spirometra mansoni* in East and Southeast Asia. The plerocercoid larvae sometimes invade the encephalon, resulting in severe cerebral sparganosis. Surgical removal of the larvae is considered a standard therapy for cerebral sparganosis. In contrast, the efficacy and safety of long-term, high-dose praziquantel treatment for cerebral sparganosis have not been explored.

**Methodology/Principal findings:**

In this multicenter retrospective study, we assessed the records of 96 patients with cerebral sparganosis who consulted at three medical centers from 2013 to 2017. Forty-two patients underwent surgical lesion removal, and the other 54 patients received long-term, high-dose praziquantel (50 mg/kg/day for 10 days, repeated at monthly intervals). The primary outcome was the complete disappearance of active lesions on cerebral magnetic resonance imaging. The secondary outcomes included the modified Rankin scale score at 90 days, incidence of seizure, eosinophil count, and serological *Spirometra*. *mansoni* antibody titer. The efficacy of praziquantel treatment was similar to that of surgical lesion removal for cerebral sparganosis with respect to both the primary outcome and secondary outcomes. Although binary logistic regression models also supported the primary outcome after adjustment for age, sex, lesion location, and loss to follow-up, some unavoidable confounders might have biased the statistical power. No significant clinical complications or laboratory side effects occurred in the praziquantel group with the exception of a relatively benign allergic reaction.

**Conclusions/Significance:**

In this small-sample, nonrandomized, retrospective exploratory study, some patients with cerebral sparganosis were responsive to long-term, high-dose praziquantel with an efficacy similar to that of surgical lesion removal. These findings increase the treatment flexibility for this serious infection.

## Author summary

Sparganosis is most prevalent in developing countries in East and Southeast Asia, probably because public health strategies have not prioritized its prevention. The plerocercoid larvae of *Spirometra mansoni* sometimes invade the brain parenchyma, resulting in cerebral sparganosis. In general, surgical removal of the larvae is considered a standard therapy for cerebral sparganosis. One alternative treatment for sparganosis is short-term, low-dose praziquantel, which has had limited success. However, the efficacy and safety of long-term, high-dose praziquantel treatment for cerebral sparganosis have not been explored. In this study, we evaluated the effectiveness of long-term, high-dose praziquantel for treatment of cerebral sparganosis in China. We conducted a retrospective exploratory study using routinely recorded data from 96 patients at three medical centers. Forty-two patients underwent surgical lesion removal, and the other 54 patients received long-term, high-dose praziquantel. Treatment of cerebral sparganosis by long-term, high-dose praziquantel showed an efficacy similar to that of surgical lesion removal with respect to the primary outcome (complete disappearance of the active lesions on cerebral magnetic resonance imaging). However, this was a small-sample, nonrandomized retrospective study, and the results should be further confirmed by a large-sample prospective study or other studies.

## Introduction

Sparganosis is a type of parasitic zoonosis associated with infection by the larval cestode of *Spirometra mansoni* [1]. Most cases have been reported in East and Southeast Asian countries, especially in China [2], South Korea [3], Japan [4], and Thailand [5]. Humans become infected with *Spirometra mansoni* by drinking water contaminated with procercoid-infected copepods, eating undercooked meat of snakes or frogs infected with *Spirometra mansoni*, or applying the flesh or skin of an infected frog or snake to poultice open wounds [1, 6]. The plerocercoid larva usually affects the subcutaneous tissue or muscle in a human host [7]. However, it can also sometimes invade the encephalon, resulting in severe cerebral sparganosis [8, 9], which can be manifested as headache, seizure, limb paralysis, aphasia, cognitive disorder, and other focal neurological deficits [10, 11]. Characteristic magnetic resonance imaging (MRI) findings such as aggregated ring-like enhancement, the tunnel sign, and wandering lesions are very useful in the diagnosis of cerebral sparganosis [12].

Although surgical removal of the parasite has been considered standard therapy [13], failure of this treatment in some cases has also been reported [14, 15]. Surgical lesion removal has been considered the first-line therapy for cerebral sparganosis just because treatments with anthelmintics, including praziquantel, have been described as ineffective [16, 17]. Since praziquantel was first introduced as a broadspectrum antiparasitic drug in 1975, it has been proven to be a successful treatment for the majority of human infections by trematodes and cestodes including schistosomiasis, clonorchiasis, paragonimiasis, taeniasis, and cysticercosis [18]. However, the efficacy of praziquantel therapy for cerebral sparganosis remains controversial. Some patients who received conventional-dose praziquantel (25mg/kg/day for 3 days) alone experienced a worsening of their clinical condition [19].

In 2012, our group reported that three patients with cerebral sparganosis showed good therapeutic responsiveness to high-dose praziquantel (50 mg/kg/day for 10 days) [9]. In 2013, Roman *et al*. also found that high-dose praziquantel treatment (75 mg/kg/day for 7 days) was efficacious in a patient with inoperable cerebral sparganosis [20]. Cerebral sparganosis is a severe and disabling disease, but public health strategies have not prioritized its prevention and treatment. Therefore, investigation of the efficacy and safety of high-dose praziquantel treatment for cerebral sparganosis is important because the drug treatment may be convenient and cost-effective. In this study, we compared the efficacy and safety of long-term, high-dose praziquantel with surgical removal in the treatment of cerebral sparganosis. Our results showed that most patients with praziquantel therapy achieved favorable outcomes during follow-up.

## Materials and methods

### Ethics statement

All patient data were anonymized in the manuscript and database. Because of the uncertainty of the efficacy and safety of long-term, high-dose praziquantel treatment for cerebral sparganosis, the routine clinical procedure was evaluated and approved by the institutional ethics review board of the First Affiliated Hospital of Nanchang University (approval number: 2013–06).

### Study design

This was a multicenter retrospective study using data collected by physicians monitoring patients with cerebral sparganosis in three academic medical centers: the First Affiliated Hospital of Nanchang University, Peking University People’s Hospital, and Jiangxi Provincial Institution of Parasitic Diseases. The primary objective of this research was to evaluate the efficacy and safety of long-term, high-dose praziquantel treatment (50 mg/kg/day for 10 days, repeated at monthly intervals) for cerebral sparganosis compared with surgical therapy. Because of the successful experience in our clinical centers before 2012 [9], we established a routine clinical procedure for cerebral sparganosis under the supervision of Drs. Hong, Xie, and Wan in the three medical centers at the end of 2012. Therefore, the clinical charts and imaging data were relatively complete in the retrospective review, but a quantitative assessment of the pretreatment disease severity was absent.

### Patient grouping

All consecutive patients with cerebral sparganosis from January 2013 to December 2017 were retrospectively retrieved from the database. The patient list was compiled by searching the electronic medical records using the International Classification of Diseases Tenth Revision (ICD10) discharge code B70.151. The inclusion criteria included: (1) patients showed cerebral symptoms associated with at least one structural lesion; (2) patients had definite evidence of sparganum infection that was proven by immunopositivity to *Spirometra mansoni* antibody in both serum and cerebrospinal fluid (CSF) tests and/or pathological evidences; (3) patients underwent follow-up cerebral MRI and serological immunological tests in our centers. The exclusion criteria included: (1) patients lost to follow-up; (2) patients with severe cardio-pulmonary dysfunction, resulting in contra-indication for surgery; (3) patients with severe liver and/or renal dysfunction, resulting in contra-indication for surgery or praziquantel treatment; (4) patients with surgical lesion removal, who had initially received praziquantel; (5) patients with praziquantel formula treatment after surgical lesion removal.

The patients were classified into a surgical therapy group and long-term, high-dose praziquantel therapy group according to the following principles. All patients initially received a detailed clinical assessment, followed by medical education about cerebral sparganosis in order to enable an informed decision regarding the choice of either praziquantel treatment or surgical treatment themselves. The physicians did not make a decision regarding treatment options for them, except patients with multiple lesions who would directly receive praziquantel treatment. The final treatment plan was chosen according to the willingness of the patients or their legal guardians. The disease severity and high-risk lesions located at some important brain regions might have been overemphasized in some communications, but the proportion could not be determined in the retrospective study. However, high-risk lesions were not considered an operative contra-indication because our neurosurgeons had excellent technical skills and experience for the operation. The main reasons for opting for long-term, high-dose praziquantel treatment were refusal to undergo surgery, lesions located at important functional areas of the brain, and initial tentative therapy (i.e., some patients wanted to try two cycles of praziquantel treatment, and subsequently underwent surgical lesion removal if a positive therapeutic outcome was not obtained).

### Variable evaluation

The clinical variables evaluated in this study were age, gender, epidemiological history, headache, seizure, hemiparesis, and aphasia. The epidemiological history was judged by whether the patients had been infected with *Spirometra mansoni* by drinking water contaminated with procercoid-infected copepods, eating undercooked meat of snakes or frogs infected with *Spirometra mansoni*, or applying the flesh or skin of an infected frog or snake to poultice open wounds. The laboratory variables were the cerebral MRI characteristics (aggregated ring-like enhancement, the tunnel sign, lesion migration, high-risk lesions, and multiple lesions), blood eosinophil percentage, and serological and CSF levels of antibodies for a panel of parasitic infections including *spirometra mansoni*, *schistosoma japonicum*, cysticercosis, paragonimiasis, clonorchiasis, toxoplasmosis, and echinococcosis. Aggregated ring-like enhancement refers to conglomerate ring-shaped enhancing lesions on MRI, usually three to six bead-shaped rings (S1A Fig). The tunnel sign is about 4 cm in length (usually 2–6 cm) and 0.8 cm in width (usually 0.5–1.5 cm), and exhibits marked enhancement on coronal and sagittal contrast MRI (S1B Fig). Lesion migration indicates the presence of new and old lesions in different cerebral locations due to the migration of larva (S1C and S1D Fig). Multiple lesions are defined as the existence of at least two active lesions located at different encephalic regions. A high-risk lesion is a lesion located at an important functional area of the brain, including the brain stem, thalamus, and precentral gyrus. The *Spirometra mansoni* IgG antibody titer was expressed as the optical density value on microplate enzyme-linked immunosorbent assay. The cut-off value of the optical density was 0.30 as determined by normal human serum in our laboratory.

### Therapeutic strategy

Surgical removal of lesions included two types of clinical procedures: craniotomy and CT-guided stereotactic aspiration. After the surgery, the patients were routinely administered with low-dose praziquantel (50 mg/kg/day for 4 days) to prevent possible residual infection. In the praziquantel treatment group, the patients were initially treated with 50 mg/kg/day in three divided doses for 10 days, and the treatment cycle was then repeated at monthly intervals, until the active lesions had completely disappeared on MRI. The MRI scan was performed at the beginning of the next treatment cycle. The maximum number of repetitive cycles of praziquantel administration could not exceed eight cycles. If more than eight were needed, the patient would be treated by surgery. If a patient developed an allergic or hypersensitive reaction during therapy course, 5 mg/day of dexamethasone was intravenously administered for no more than five days.

### Efficacy assessments

The clinical manifestations of cerebral sparganosis were closely related to the site of the lesions, and the clinical prognosis was significantly associated with the recovery of granulomatous lesions. Active ring-like or tunnel-like enhancements represented the direction of active inflammatory tunnels in different dimensions. Therefore, the primary efficacy endpoint was defined as the disappearance of active lesions on contrast MRI. All patients underwent an MRI scan before the treatment. The surgical patients underwent a follow-up MRI at one month postoperatively. The patients treated by praziquantel underwent an MRI scan at the beginning of each treatment cycle. If the active lesions disappeared, the praziquantel treatment was discontinued. If a patient developed neurological symptoms at any time after completion of the treatment regimen, cerebral MRI was performed immediately. Development of a new lesion after the active lesions had disappeared was defined as treatment failure.

The secondary efficacy endpoints were the clinical outcome assessed by the modified Rankin scale (mRS) score 90 days after the end of the treatment, the incidence of seizure, the eosinophil count, and the serological *Spirometra mansoni* antibody titer. The mRS is a 7-point scale ranging from 0 (no symptoms) to 6 (death). A score of ≤2 indicates functional independence. A seizure was defined as a clinical event occurring after the end of treatment regardless of whether seizures existed before treatment. The eosinophil count was determined at the end of treatment, and the number of patients with an eosinophil percentage of >5% was counted. Serological titers were only compared between the beginning and the end of treatment.

### Safety assessments

The clinical symptoms assessed in this study were vital signs, headache, dizziness, sleepiness, abdominal pain, diarrhea, and any reported adverse events. The laboratory indices were hematology parameters, alanine aminotransferase, aspartate aminotransferase, blood creatinine, blood urea nitrogen, urine parameters, and electrocardiography. An allergy to praziquantel treatment for cerebral sparganosis usually manifested as fever, chills, pruritus, and urticaria occurred in the first or second treatment cycle. Adverse events were defined as clinical symptoms with onset or worsening severity at or after the first dose of praziquantel until the end of the safety follow-up (day 30).

### Statistical analysis

All statistical analyses were performed using the Statistical Package for the Social Sciences 17.0 software (SPSS, Inc., Chicago, IL, USA), and a p-value of <0.05 was considered statistically significant. Categorical variables were presented as count (percentage). Continuous variables were reported as mean ± standard deviation. The statistical significance of intergroup differences was assessed by pooled-variance and separate-variance Student’s t-test, chi-squared test, or Fisher’s exact test as appropriate. To adjust for the confounders in this retrospective study, several binary logistic regression models were established in a sub-analysis to identify the outcomes.

## Results

### Patient demographic and baseline characteristics

**Fig 1:**
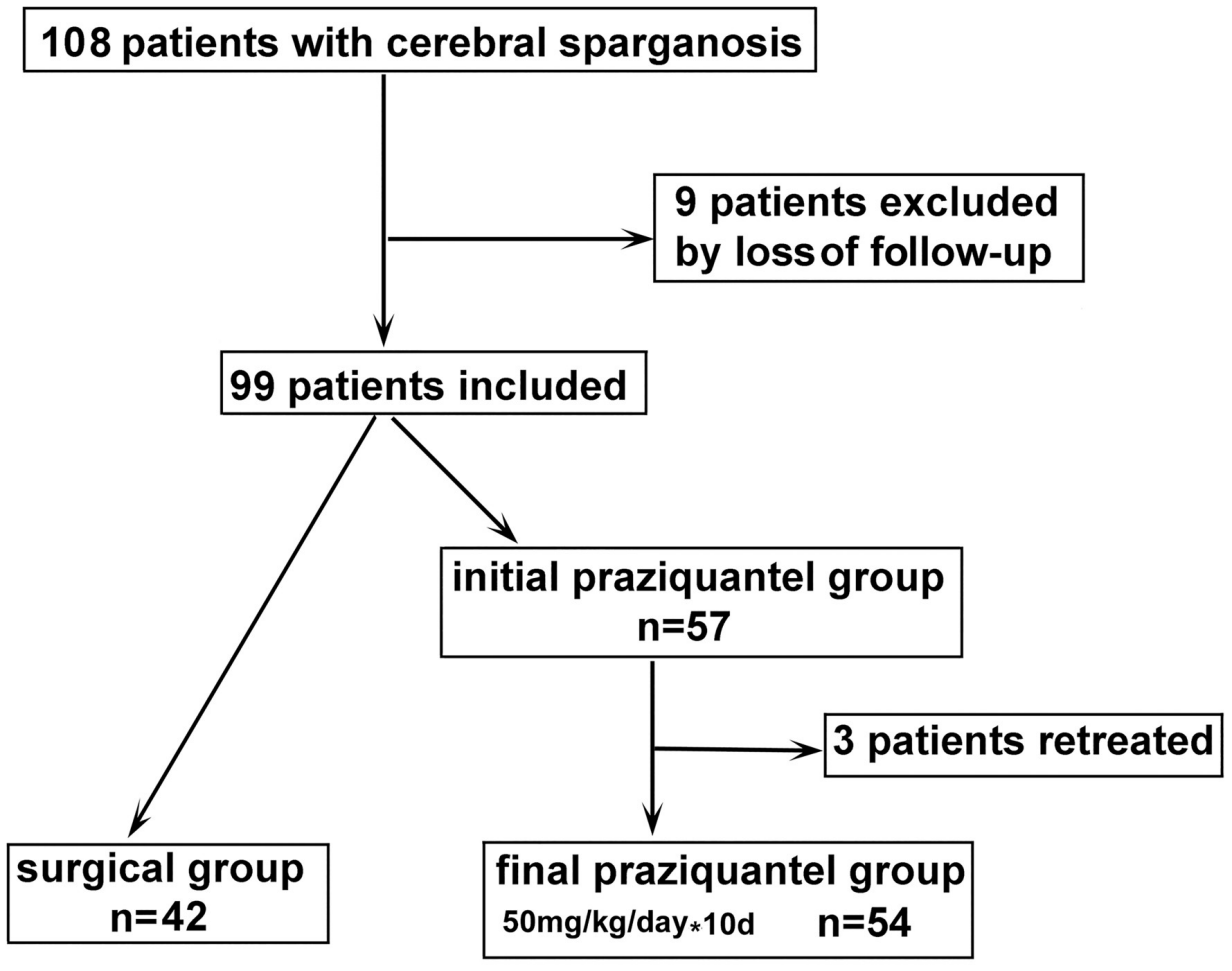
Flowchart of study enrollment and rationale for exclusion.

The patient disposition and analysis were depicted in Fig 1. In total, 108 patients were screened by the discharge code, among whom 99 patients met the inclusion criteria and nine were excluded due to loss to follow-up. Initially, 42 patients underwent surgical lesion removal, and 57 patients received long-term, high-dose praziquantel. Three patients who initially received tentative praziquantel treatment underwent surgery when no therapeutic effects were observed after two treatment cycles. Therefore, the surgical group comprised 42 patients and the praziquantel group comprised 54 patients. There were no significant differences in demographics, epidemiological history, clinical manifestations, or relevant laboratory data between the two groups (Table 1 and S1 Table).

**Table 1:** Baseline characteristics between the praziquantel and surgical treatment groups.

Variables	Praziquantel group (n = 54)	Surgical group (n = 42)	p value^a^
**Age (years)**	28.02±16.67	28.88±12.77	0.784
**Male**	37 (68.5%)	33 (78.6%)	0.272
**Clinical features**			
Epidemiological history	10 (18.5%)	7 (16.7%)	0.814
Headache	21 (38.9%)	14 (33.3%)	0.575
Seizure	40 (74.1%)	32 (76.2%)	0.812
Hemiparesis	22 (40.7%)	18 (42.9%)	0.727
Aphasia	11 (20.4%)	9 (21.4%)	0.899
**Radiological changes**			
Ring-like enhancement	53 (98.1%)	41 (97.9%)	1.000
Tunnel sign	45 (83.3%)	32 (76.2%)	0.384
Lesion migration	13 (24.1%)	10 (23.8%)	0.976
Multiple lesions	5 (9.3%)	0 (0%)	0.066
High-risk lesion	7 (13.0%)	4 (9.5%)	0.751
**Laboratory tests**			
Eosinophil percentage >5%	30 (55.6%)	22 (52.4%)	0.757
Serological titer (OD)	1.81±0.83	1.65±0.81	0.355
CSF titer (OD)	1.25±0.47	1.13±0.53	0.240

Abbreviation: OD, optical density; CSF, cerebrospinal fluid.

^a^ Comparison between the two groups, using pooled-variances Student’s t test, chi-squared test, or Fisher’s exact test as appropriate, except for age comparison using separate-variances Student’s t-test.

### Clinical outcomes

All 42 patients in the surgery group initially underwent surgical lesion removal (24 by craniotomy and 18 by CT-guided stereotactic aspiration) and achieved complete lesion recovery one month after surgery. However, three patients developed new lesions during the postoperative follow-up: one had a new lesion in the second month after stereotactic aspiration, one reoccurred in the third month after craniotomy, and one reoccurred in the fifth month after stereotactic aspiration. The treatment effects between craniotomy and CT-guided stereotactic aspiration were not significantly different (95.8% vs 88.9%; absolute difference, 6.9%; 95% confidence interval [-23.9%, 36.9%]; p = 0.567; Fisher’s exact test).

**Fig 2:**
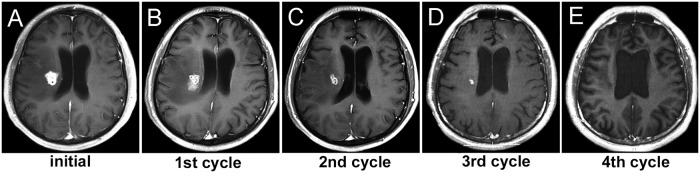
Dynamic cerebral MRI changes after praziquantel treatment. A 46-year-old male patient presented with left hemiplegia for three weeks and was diagnosed as cerebral sparganosis. He was treated with praziquantel (50mg/kg/day) in three divided doses for 10 days, and then the treatment cycle was repeated with monthly intervals until the active lesion completely disappeared on enhanced MRI. The initial enhanced MRI showed the aggregated ring-like lesions combined with severe edema at the right of periventricular (A). After the first treatment cycle, the lesion reduced a little in size (B). After the second treatment cycle, the lesion was obviously smaller (C). After the third treatment cycle, the lesion showed a resolution of swelling (D). After the fourth treatment cycle, the lesion had completely disappeared (E).

Fifty-one patients who underwent long-term, high-dose praziquantel treatment achieved complete lesion recovery at the end of treatment. Three patients still had active lesions after eight cycles of praziquantel treatment, and then underwent surgical removal. Among the 51 patients, three had recurrent lesions at the third, fourth, and sixth month after recovery of the active lesions, respectively. Among the patients who achieved successful praziquantel treatment, two underwent one treatment cycle; five underwent two treatment cycles; 19 underwent three treatment cycles; 13 underwent four treatment cycles (Fig 2); and nine required five to eight treatment cycles (S2 Table and S2 Fig). The long-term, high-dose praziquantel treatment for cerebral sparganosis showed an efficacy similar to that of surgical lesion removal with respect to the primary efficacy endpoint (88.9% vs. 92.9%; p = 0.727) (Table 2). Even when the cut-off of the number of treatment cycles was set at five, the primary efficacy endpoint still showed no significant difference between the praziquantel and surgical groups (81.5% vs 92.9%; p = 0.106). No patients in either group had a mRS score of more than or equal to 5. The number of patients with a mRS score of ≤2 was similar between the praziquantel and surgical groups. The incidence of seizures after the end of treatment was similar between the praziquantel and surgical groups. The numbers of patients with an eosinophil count of >5% and the serological *Spirometra mansoni* antibody titer were similar between the two groups at the end of treatment (Table 2).

**Table 2:** Results of the primary and secondary efficacy outcomes between the praziquantel and surgical groups.

Outcomes	Praziquantel group (n = 54)	Surgical group (n = 42)	p value	Absolute Diff [95% CI]
**Primary outcome**				
No active lesions after eight cycles	48 (88.9%)	39 (92.9%)	0.727	-4.0%[-23.9%, 16.2%]
No active lesions after five cycles	44 (81.5%)	39 (92.9%)	0.106^a^	-11.4%[-24.3%, 1.6%]
**Secondary outcome**				
mRS at 90 days			0.726	2.1%[-18.0%, 22.1%]
0–2	50 (92.6%)	38 (90.5%)		
3–4	4 (7.4%)	4 (9.5%)		
Incidence of seizure	8 (14.8%)	8 (19.0%)	0.581^a^	-4.2%[-19.4%, 11.0%]
Eosinophil count (>5%)	1 (1.9%)	1 (2.4%)	1.000	-0.5%[-20.6%, 20.0%]
Serological titer (OD)	0.18±0.14	0.20±0.13	0.704	-0.021[-0.077, 0.049]

Abbreviation: mRS, modified Rankin scale; OD, optical density; CI, confidence interval.

^a^ Comparison between the two groups, using Student’s t test or Fisher’s exact test, except active lesion recovery with cut-off of five cycles and incidence of seizure using chi-squared test.

Several logistic regression models were configured to further evaluate the confounders that may have introduced bias into this retrospective study. After adjustment for age, sex, multiple lesions, and high-risk lesions, the long-term, high-dose praziquantel treatment showed an efficacy similar to that of surgical lesion removal (Table 3 and S4 Table). Because the lesion locations was considered to be a possible major confounder causing selection bias, the patients with multiple lesions and high-risk lesions were excluded from a sub-analysis, which showed that the long-term, high-dose praziquantel treatment still had efficacy similar to that of surgical removal (Table 3). Of the nine patients who were lost to follow up and thus initially excluded from the study, six had not received any treatments after diagnosis, but the other three had initially received praziquantel for two to three cycles. Even when these three patients plus the three retreated patients were counted as a negative primary outcome in the praziquantel group, the sub-analysis adjusted for age, sex, multiple lesions, and high-risk lesions showed no significant difference between the praziquantel and surgical groups (Table 3). Considering that allergic reactions were only observed in the praziquantel group, the patients with allergic reactions were excluded from the sub-analysis, which still showed that the praziquantel treatment had an efficacy similar to that of surgical removal (Table 3).

**Table 3:** Sub-analysis of primary outcome by binary logistic regression models.

Sub-analysis	Praziquantel group (m/n)	Surgical group (m/n)	p value	OR [95% CI]
primary outcome unadjusted	48/54	39/42	0.727	1.625 [0.382, 6.920]
adjusted by two variables ^a^	48/54	39/42	0.532	1.592 [0.371, 6.840]
adjusted by four variables ^b^	48/54	39/42	0.441	1.778 [0.411, 7.072]
groups without multiple lesions ^a^	43/49	39/42	0.447	1.764 [0.409, 7.616]
groups without multiple lesions and high-risk lesion ^a^	36/42	36/38	0.221	2.857 [0.532, 15.344]
groups including additional patients ^b^*	48/60	39/42	0.105	3.091 [0.791, 12.072]
groups without allergic patients ^b^^#^	42/48	39/42	0.358	1.989 [0.459, 8.615]

Abbreviation: OR, odds ratio; CI, confidence interval; m, number of effective cases; n, total of case number.

^a^, adjusted by age and gender;

^b^, adjusted by age, sex, multiple lesions, and high-risk lesion

*, Three patients retreated and three patients lost to follow up were counted as negative primary outcome in the praziquantel group.

^#^, Six patients with allergic reaction were excluded from the praziquantel group.

### Safety and tolerability

Six patients developed allergic reactions in the praziquantel group, but no patients developed allergic reactions in the surgical group. Although a difference was observed in the number of patients with allergic reactions between the two groups (Table 4), the clinical course of the allergic reactions was relatively benign and rapidly resolved after the administration of 5mg/day of dexamethasone. Headache, dizziness, abdominal pain, diarrhea, and sleepiness were also reported by the patients. The incidence of these symptoms was similar between the praziquantel and surgical groups (Table 4). No differences in vital signs or electrocardiography findings were identified between the two groups. Increases in the aspartate aminotransferase (AST) and alanine aminotransferase (ALT) indicated a possibility of liver dysfunction, which occurred in a small proportion of the patients in the first or second treatment cycle. The dysfunction was characterized by two- to four- fold elevation of the AST/ALT without jaundice and was resolved by symptomatic treatment. Overall, no clinically meaningful differences in these laboratory abnormalities were observed between the two groups (Table 4). No patients withdrew from the study because of adverse events. Binary logistic regression models adjusted for age, sex, multiple lesions, and high-risk lesion further showed that these safety variables were not different between the two groups (S5 Table).

**Table 4:** Comparison of adverse events between the praziquantel and surgical groups.

Adverse events	Praziquantel group (n = 54)	Surgical group (n = 42)	p value
**Clinical events**			
Allergic reaction	6 (11.1%)	0 (0.0%)	0.034
Headache	10 (18.5%)	8 (19.0%)	1.000^a^
Dizziness	6 (11.1%)	3 (7.1%)	0.508^a^
Sleepiness	4 (7.4%)	1 (2.4%)	0.382
Abdominal pain	4 (7.4%)	0 (0.0%)	0.129
Diarrhea	3 (5.6%)	1 (2.4%)	0.629
**Laboratory events**			
ALT increase	5 (9.3%)	1 (2.4%)	0.226
AST increase	6 (11.1%)	1 (2.4%)	0.132
Creatinine increase	3 (5.6%)	2 (4.8%)	1.000
BUN increase	2 (3.7%)	0 (0.0%)	0.503
Proteinuria	1 (1.9%)	0 (0.0%)	1.000

Abbreviation: ALT, alanine aminotransferase; AST, aspartate aminotransferase; BUN, blood urea nitrogen.

^a^ Comparison between the two groups, using Student’s t test or Fisher’s exact test, except for headache and dizziness using chi-squared test.

## Discussion

Cerebral sparganosis is a severe disease when the plerocercoid larva of *Spirometra* tapeworm targets the central nervous system [21]. The larva damages brain tissue and gives rise to neurological function deficits caused by inflammatory attacks and the migration process [22, 23]. The natural lifetime of the larva is several decades long in some cases [24]. Therefore, eradication of the larva is the main therapeutic strategy [25]. Surgical removal is considered the first-line treatment for cerebral sparganosis because treatment with anthelmintics, including praziquantel, has been described as ineffective [16, 17]. However, both craniotomy and CT-guided stereotactic aspiration may result in incomplete removal, especially when remnants of the scolex segment are left behind after CT-guided stereotactic aspiration, resulting in larva regeneration [16]. Additionally larva lesions may localize at some important functional structures, in which cases the surgical procedure will cause severe neurological dysfunction. Thus, the surgical removal of larva has some limitations.

In this study, long-term, high-dose praziquantel resolved the cerebral lesions in 88.9% of patients with cerebral sparganosis. Most patients with cerebral sparganosis achieved effective treatment by praziquantel at 50 mg/kg/day for 10 days at the third or fourth treatment cycle. The absolute efficacy rate of praziquantel was relatively lower than that of surgical removal, but there were no significant differences in the primary efficacy endpoint (no active lesions on MRI) and secondary efficacy endpoints (mRS score at 90 days, seizure, eosinophil count, and serological titer) between the praziquantel and surgical groups. Quantitative assessments of disease severity before therapy were unavailable in this retrospective study. Naturally, it was possible that patients with milder severity whose clinical course showed little progression and who may not need invasive surgery might select praziquantel treatment. In addition, patients with complicated clinical course or multiple or high-risk lesions might not select surgical removal. As a result, although there were no significant differences in the available baseline data between the praziquantel and surgical groups, some confounders could have biased the primary outcome. To compensate for this bias, several logistic regression models were established to adjust for age, sex, lesion location, and loss to follow-up. These regression models showed that praziquantel still had an efficacy similar to that of surgery, but some of these results actually showed borderline significance and indicated a tendency toward a surgical benefit.

Praziquantel can significantly damage and destroy the whole body of the plerocercoid except the scolex and neck. The spargana may regenerate from the remnants of the scolex and neck, which might be responsible for resistance to praziquantel [26]. Therefore, the therapeutic efficacy of praziquantel for human sparganosis remains controversial. In one study, a patient with subcutaneous sparganosis failed to recover after being treated with two praziquantel regimens of 3×25mg/kg×5 days at one month interval [27]. In contrast, patients with pleural or pericardial sparganosis were successfully cured with a regimen of 60 to 75 mg/kg/day of praziquantel for three days [27,28]. For sparganosis in the central nervous system, praziquantel treatment might be more difficult because only 1/7 to 1/5 of the drug within the plasma can diffuse into the tissue near the larva lesions [29]. Previous studies also supported the finding that praziquantel treatment dose not seem to have a killing effect on live worms [19]. In a recent study, however, the larva in a patient with cerebral sparganosis was successfully eradicated by high-dose praziquantel with a regimen of 3×25 mg/kg/day for seven days combined with cimetidine (3×400mg daily) and a high-carbohydrate diet [20]. As early as 2012, we modified our praziquantel treatment formula to 50 mg/kg/day in three divided doses for 10 days, and then repeated this cycle at monthly intervals until the active lesions completely disappeared on enhanced MRI. Our clinical data showed that most patients with cerebral sparganosis were effectively treated with three or four treatment cycles. The host’s immune cells (e.g., granulocytes, histiocytes) and antibodies might act synergistically with praziquantel in the treatment of tissue-invading sparganosis infections [30]. The repeated administration of praziquantel might increase the probability of antigen exposure on the surface of the regenerative worm, which might produce more immunoreactivity to attack the regenerative larva [31]. However, the detailed pharmacological mechanism needs to be investigated in further studies.

It is generally acknowledged that praziquantel is highly safe without serious adverse reactions. Common adverse reactions to praziquantel are usually mild and include abdominal pain, diarrhea, dizziness, sleepiness, and headache [32]. These clinical complications were not significantly different between the praziquantel and surgical groups of the present study. In rare situations, however, praziquantel might cause allergic or hypersensitive reactions in some patients [33]. In the present study, these allergic reactions usually occurred in the first or second treatment cycle with a benign clinical course, and were sensitive to short-term, low-dose dexamethasone. Although some patients showed a tendency toward mild liver dysfunction in the first two praziquantel treatment cycles, the laboratory indices showed no significant differences between the two groups. Importantly, follow-up of the patients treated with long-term, high-dose praziquantel revealed no delayed adverse events.

This study had some limitations that need to be explicitly acknowledged. First, it was a small-sample, nonrandomized retrospective study; thus, the statistical power was low. Cases of cerebral sparganosis are rare, so it is necessary to include data from more medical centers. Second, the fact that the patients made the treatment decision themselves undermined the reliability of the results. Confounders such as the disease severity, multiple lesions, high-risk lesions, and doctor-patient communication skills might have produced some bias with respect to the treatment decision. Third, the clinical data were quite heterogeneous because the treatment with praziquantel was different in each patient based on the lesion site and response. It was impracticable to assess the outcome only once and at a fixed time to avoid biasing the results because of ethical considerations and the pharmacological properties of praziquantel. Overall, the outcomes of this study should be cautiously interpreted that long-term, high-dose praziquantel had an efficacy similar to that of surgical removal, though the logistic regression models supported these results. Indeed, several baseline outcomes and safety indices showed borderline significance with respect to the benefit of surgical removal of larva lesion. However, the findings in this exploratory study involving real-world practitioners with a high level of experience add treatment flexibility for this serious infection, and provide a basis to promote a large-sample, randomized, prospective study of long-term, high-dose praziquantel treatment for cerebral sparganosis in the future.

## Supporting information

S1 Fig Typical MRI characteristics for cerebral sparganosis. Aggregated ring-like enhancement indicates a conglomerated ring-like enhancement, which is seen as bead shaped, usually four to six rings, on MRI (A). The tunnel sign is about 4 cm in length (usually 2–6 cm) and 0.8 cm in width (usually 0.5–1.5 cm), which shows marked enhancement on coronal and sagittal contrast MRI (B). Lesion migration indicates the presence of new (C) and old (D, seven months ago) lesions in different cerebral locations.
(TIF)

S2 Fig The distribution of patients received praziquantel cycles. Among patients with successful praziquantel treatment, two patients underwent one praziquantel cycle; five patients underwent two praziquantel cycles; 19 patients underwent three praziquantel cycles; 13 patients underwent four praziquantel cycles; five patients underwent five praziquantel cycles; two patients underwent six praziquantel cycles; one patient underwent seven praziquantel cycles; one patient underwent eight praziquantel cycles.
(TIF)

S3 Fig The lesion location in the group and surgical groups. No differences were found across the 2 groups.
(TIF)

S1 Table The demographic and baseline data between the praziquantel and surgical groups. (XLS)

S2 Table The clinical outcomes data between the praziquantel and surgical groups. (XLS)

S3 Table The safety and tolerability between the praziquantel and surgical groups. (XLS)

S4 Table Logistic regression models for clinical outcomes adjusted by age, sex, multiple lesions, and high-risk lesion. (DOC)

S5 Table Logistic regression models for safety variables adjusted by age, sex, multiple lesions, and high-risk lesion. (DOC)

S1 Checklist STROBE checklist. (DOC)

## References

[1] Liu Q, Li MW, Wang ZD, Zhao GH, Zhu XQ. Human sparganosis, a neglected food borne zoonosis. Lancet Infect Dis. 2015;15:1226–35. 10.1016/S1473-3099(15)00133-4 . 26364132

[2] Li MW, Song HQ, Li C, Lin HY, Xie WT, Lin RQ, et al Sparganosis in mainland China. Int J Infect Dis. 2011;15:e154–6. 10.1016/j.ijid.2010.10.001 . 21126898

[3] Chang KH, Chi JG, Cho SY, Han MH, Han DH, Han MC. Cerebral sparganosis: analysis of 34 cases with emphasis on CT features. Neuroradiology. 1992;34:1–8. . 1553030 10.1007/BF00588423

[4] Yamashita K, Akimura T, Kawano K, Wakuta Y, Aoki H, Gondou T. Cerebral sparganosis mansoni. Report of two cases. Surg Neurol. 1990;33:28–34. . 2405529 10.1016/0090-3019(90)90221-a

[5] Anantaphruti MT, Nawa Y, Vanvanitchai Y. Human sparganosis in Thailand: an overview. Acta Trop. 2011;118:171–6. 10.1016/j.actatropica.2011.03.011 . 21459073

[6] Cui J, Wang Y, Zhang X, Lin XM, Zhang HW, Wang ZQ, et al A neglected risk for sparganosis: eating live tadpoles in central China. Infect Dis Poverty. 2017;6:58 10.1186/s40249-017-0265-7 28468685 PMC5415782

[7] Dunn IJ, Palmer PE. Sparganosis. Semin Roentgenol. 1998;33:86–8. . 9516692 10.1016/s0037-198x(98)80034-5

[8] Fan KJ, Pezeshkpour GH. Cerebral sparganosis. Neurology. 1986;36:1249–51. . 3748393 10.1212/wnl.36.9.1249

[9] Hong D, Xie H, Zhu M, Wan H, Xu R, Wu Y. Cerebral sparganosis in mainland Chinese patients. J Clin Neurosci. 2013;20:1514–9. 10.1016/j.jocn.2012.12.018 . 23911107

[10] Rengarajan S, Nanjegowda N, Bhat D, Mahadevan A, Sampath S, Krishna S. Cerebral sparganosis: a diagnostic challenge. Br J Neurosurg. 2008;22:784–6. 10.1080/02688690802088073 . 18661311

[11] Sundaram C, Prasad VS, Reddy JJ. Cerebral sparganosis. J Assoc Physicians India. 2003;51:1107–9. . 15260399

[12] Song T, Wang WS, Zhou BR, Mai WW, Li ZZ, Guo HC, et al CT and MR characteristics of cerebral sparganosis. AJNR Am J Neuroradiol. 2007;28:1700–5. 10.3174/ajnr.A0659 . 17885230 PMC8134205

[13] Kim DG, Paek SH, Chang KH, Wang KC, Jung HW, Kim HJ, et al Cerebral sparganosis: clinical manifestations, treatment, and outcome. J Neurosurg. 1996;85:1066–71. 10.3171/jns.1996.85.6.1066 . 8929496

[14] Deng L, Xiong P, Qian S. Diagnosis and stereotactic aspiration treatment of cerebral sparganosis: summary of 11 cases. J Neurosurg. 2011;114:1421–5. 10.3171/2010.4.JNS1079 . 20486898

[15] Yu Y, Shen J, Yuan Z, Xia Z, Gao F, Jiang L, et al Cerebral Sparganosis in Children: Epidemiologic and Radiologic Characteristics and Treatment Outcomes: A Report of 9 Cases. World Neurosurg. 2016;89:153–8. . 26855309 10.1016/j.wneu.2016.01.086

[16] Torres JR, Noya OO, Noya BA, Mouliniere R, Martinez E. Treatment of proliferative sparganosis with mebendazole and praziquantel. Trans R Soc Trop Med Hyg. 1981;75:846–7. . 7330947 10.1016/0035-9203(81)90428-4

[17] Sohn WM, Hong ST, Chai JY, Lee SH. Infectivity of the sparganum treated by praziquantel, gamma-irradiation and mechanical cutting. Korean J Parasitol. 1993;31:135–9. . 8343455 10.3347/kjp.1993.31.2.135

[18] Chai JY. Praziquantel treatment in trematode and cestode infections: an update. Infect Chemother. 2013;45:32–43. 10.3947/ic.2013.45.1.32 24265948 PMC3780935

[19] Chai JY, Yu JR, Lee SH, Kim S, Cho SY. Ineffectiveness of praziquantel treatment for human sparganosis: a case report. Seoul J Med. 1988; 29:397–9.

[20] Gonzenbach RR, Kong Y, Beck B, Buck A, Weller M, Semmler A. High-dose praziquantel therapy for cerebral sparganosis. J Neurol. 2013;260:1423–5. 10.1007/s00415-013-6901-7 . 23546305

[21] Chiu CH, Chiou TL, Hsu YH, Yen PS. MR spectroscopy and MR perfusion character of cerebral sparganosis: a case report. Br J Radiol. 2010;83:e31–4. 10.1259/bjr/73038348 20139254 PMC3473539

[22] Lo Presti A, Aguirre DT, De Andrés P, Daoud L, Fortes J, Muñiz J. Cerebral sparganosis: case report and review of the European cases. Acta Neurochir (Wien). 2015;157:1339–43. 10.1007/s00701-015-2466-9 . 26085111

[23] Wang P, Su X, Mao Q, Liu Y. The surgical removal of a live tapeworm with an interesting pathologic finding most likely representing the migration path: a case report of cerebral sparganosis. Clinics (Sao Paulo). 2012;67:849–51. 10.6061/clinics/2012(07)24 22892934 PMC3400180

[24] Chu S, Lu X, Wang Y, Gao G, Xv F, Zee CS, et al Magnetic resonance imaging features of pathologically proven cerebral sparganosis. J Int Med Res. 2013;41:867–77. 10.1177/0300060513480925 . 23680666

[25] Shirakawa K, Yamasaki H, Ito A, Miyajima H. Cerebral sparganosis: the wandering lesion. Neurology. 2010;74:180 10.1212/WNL.0b013e3181c91a15 20065255

[26] Chan JD, Zarowiecki M, Marchant JS. Ca^2^⁺ channels and praziquantel: a view from the free world. Parasitol Int. 2013;62:619–28. 10.1016/j.parint.2012.12.001 . 23246536 PMC3610807

[27] Ishii H, Mukae H, Inoue Y, Kadota JI, Kohno S, Uchiyama F, et al A rare case of eosinophilic pleuritis due to sparganosis. Intern Med. 2001;40:783–5. . 11518125 10.2169/internalmedicine.40.783

[28] Lee JH, Kim GH, Kim SM, Lee SY, Lee WY, Bae JW, et al A case of sparganosis that presented as a recurrent pericardial effusion. Korean Circ J. 2011;41:38–42. 10.4070/kcj.2011.41.1.38 . 21359068 PMC3040402

[29] Andrews P, Thomas H, Pohlke R, Seubert J. Praziquantel. Med Res Rev. 1983;3:147–200. . 6408323 10.1002/med.2610030204

[30] Doenhoff MJ, Cioli D, Utzinger J. Praziquantel: mechanisms of action, resistance and new derivatives for schistosomiasis. Curr Opin Infect Dis. 2008;21:659–67. 10.1097/QCO.0b013e328318978f . 18978535

[31] Hu DD, Cui J, Wang L, Liu LN, Wei T, Wang ZQ. Immunoproteomic Analysis of the Excretory-Secretory Proteins from Spirometra mansoni Sparganum. Iran J Parasitol. 2013;8:408–16. . 24454434 PMC3887242

[32] Rim HJ. The current pathobiology and chemotherapy of clonorchiasis. Kisaengchunghak Chapchi. 1986;24:1–141. . 12902642 10.3347/kjp.1986.24.suppl.1

[33] Shen C, Choi MH, Bae YM, Yu G, Wang S, Hong ST. A case of anaphylactic reaction to praziquantel treatment. Am J Trop Med Hyg. 2007;76:603–5. . 17360893

