# Supplementary figures and images for: Efficacy comparison between long-term high-dose praziquantel and surgical therapy for cerebral sparganosis: A multicenter retrospective cohort study
Source: PLoS Negl Trop Dis. 2018 Oct 22;12(10):e0006918. doi: 10.1371/journal.pntd.0006918 (PMC6211769; doi:10.1371/journal.pntd.0006918)

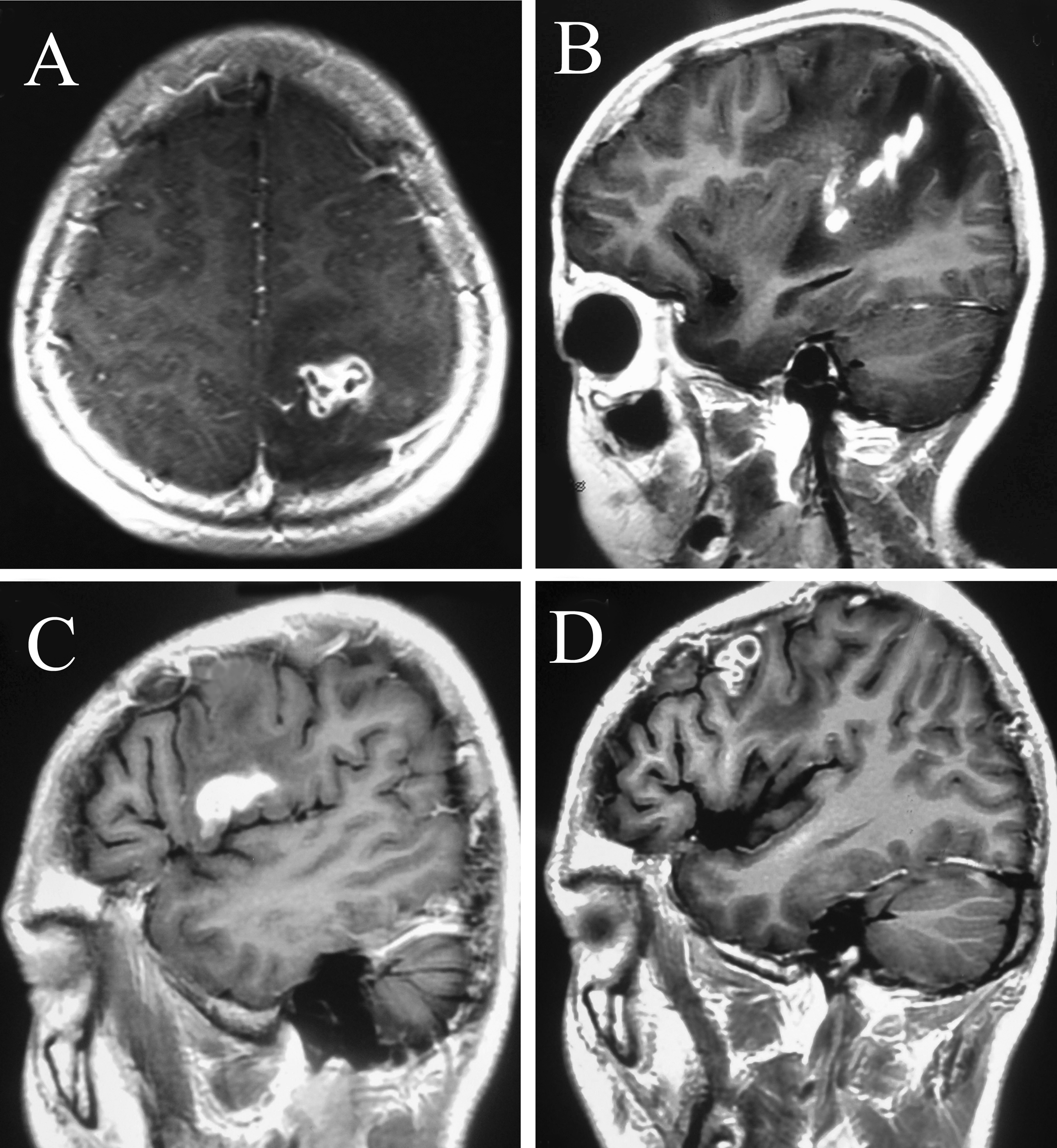

Supplement: S1 Fig — Aggregated ring-like enhancement indicates a conglomerated ring-like enhancement, which is seen as bead shaped, usually four to six rings, on MRI (A). The tunnel sign is about 4 cm in length (usually 2–6 cm) and 0.8 cm in width (usually 0.5–1.5 cm), which shows marked enhancement on coronal and sagittal contrast MRI (B). Lesion migration indicates the presence of new (C) and old (D, seven months ago) lesions in different cerebral locations. (TIF) [file pntd.0006918.s001.tif]

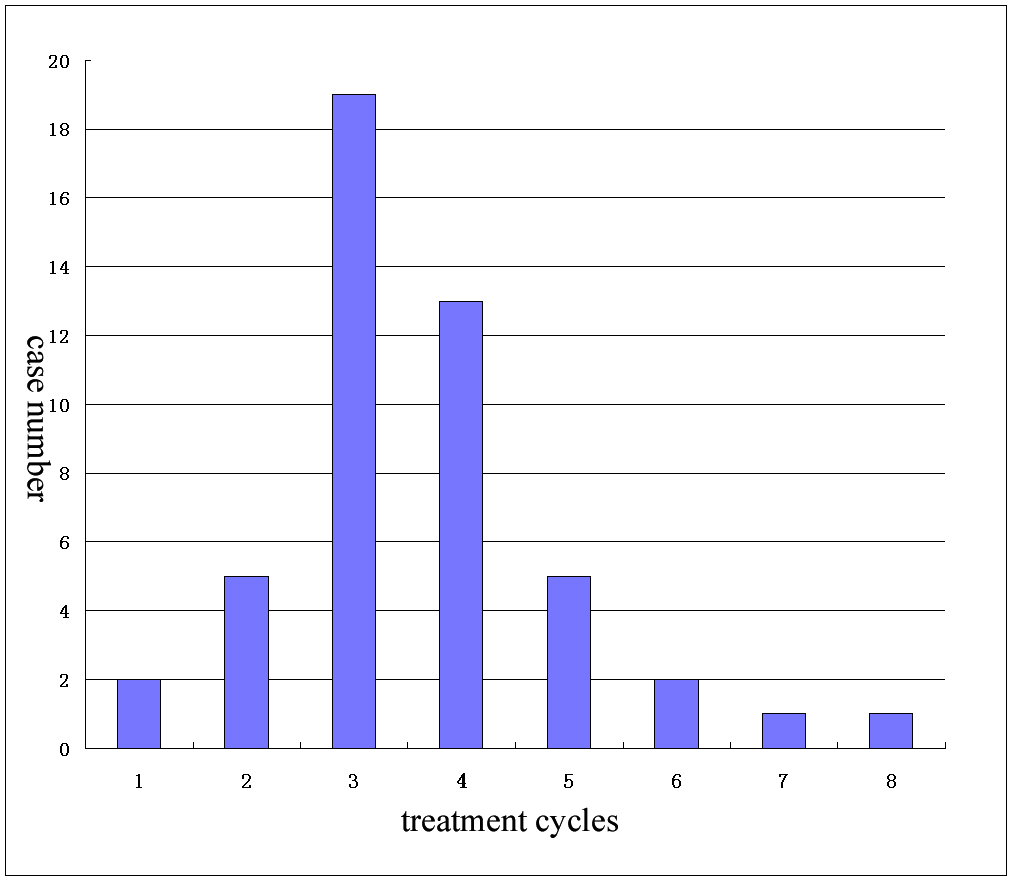

Supplement: S2 Fig — Among patients with successful praziquantel treatment, two patients underwent one praziquantel cycle; five patients underwent two praziquantel cycles; 19 patients underwent three praziquantel cycles; 13 patients underwent four praziquantel cycles; five patients underwent five praziquantel cycles; two patients underwent six praziquantel cycles; one patient underwent seven praziquantel cycles; one patient underwent eight praziquantel cycles. (TIF) [file pntd.0006918.s002.tif]

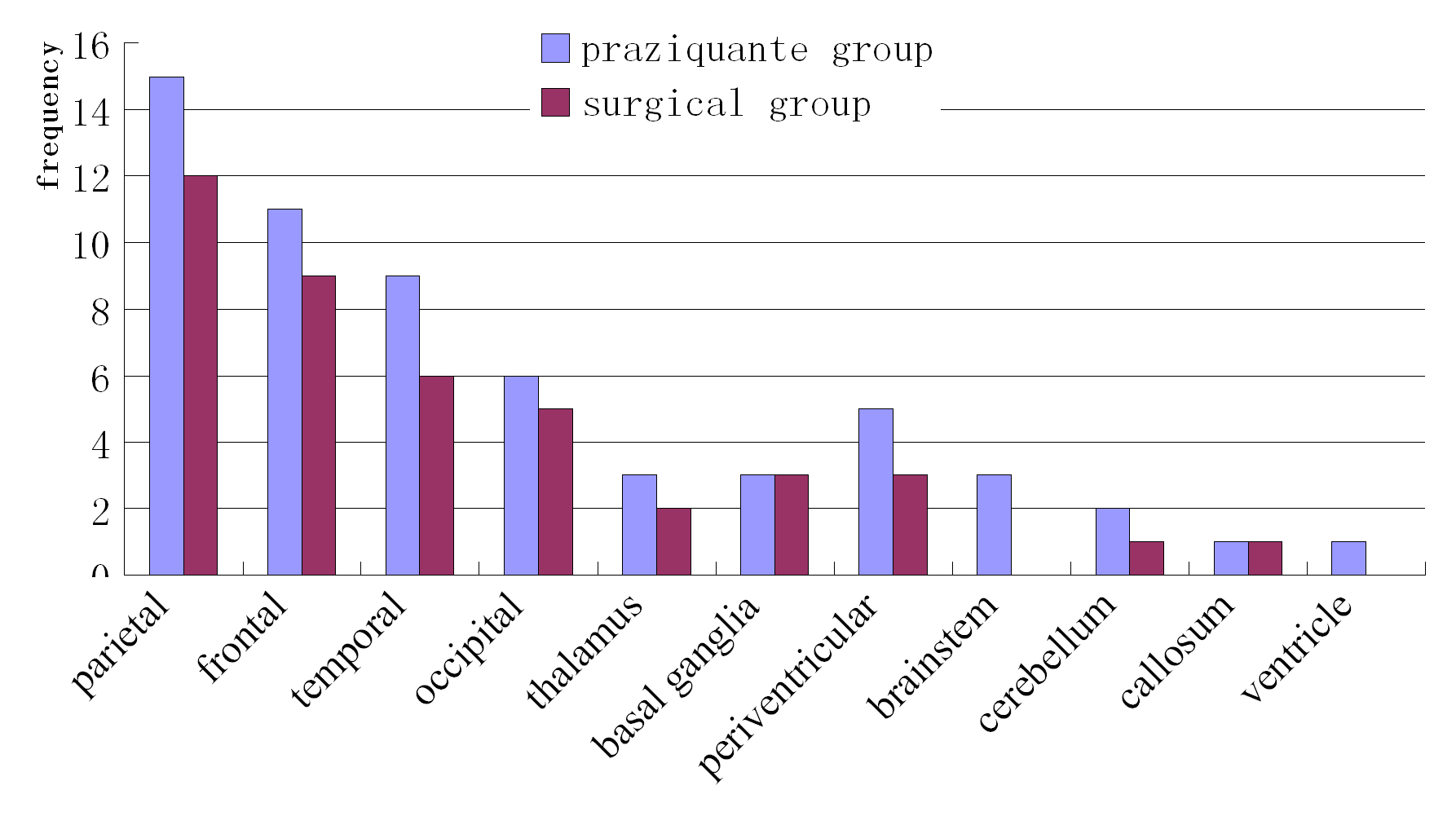

Supplement: S3 Fig — No differences were found across the 2 groups. (TIF) [file pntd.0006918.s003.tif]
