# Supplementary material for: Efficacy comparison between long-term high-dose praziquantel and surgical therapy for cerebral sparganosis: A multicenter retrospective cohort study
Source: PLoS Negl Trop Dis. 2018 Oct 22;12(10):e0006918. doi: 10.1371/journal.pntd.0006918 (PMC6211769; doi:10.1371/journal.pntd.0006918)
Supplement: S4 Table — (DOC) [file pntd.0006918.s007.doc]

**S4 Table. Logistic regression models for clinical outcomes adjusted by age, sex, multiple lesions, and high-risk lesion.**

| **Outcomes** | **Praziquantel group (n=54)** | **Surgical group (n=42)** | **OR [95% CI]** |
| --- | --- | --- | --- |
| **Primary outcome** | | | |
| No active lesions after eight cycles | 48 (88.9%) | 39 (92.9%) | 1.778 [0.411, 7.072] |
| **Secondary outcome** | | | |
| mRS at 90 days (0-2) | 50 (92.6%) | 38 (90.5%) | 1.174[0.271, 5.087] |
| Incidence of seizure | 8 (14.8%) | 8 (19.0%) | 1.255[0.419, 3.757] |
| Eosinophils count (>5%) | 1 (1.9%) | 1 (2.4%) | 1.286[0.075, 22.125] |
| Serological titer (OD) | 12 (22.2%) | 11 (26.2%) | 1.862[0.637, 5.446] |

Abbreviation: OR, odds ratio; CI: confidence interval; mRS: modified Rankin Scales; OD: optical density. Cut-off value of OD was 0.30 as determined by normal human serum in our labs.
