# Supplementary material for: Efficacy comparison between long-term high-dose praziquantel and surgical therapy for cerebral sparganosis: A multicenter retrospective cohort study
Source: PLoS Negl Trop Dis. 2018 Oct 22;12(10):e0006918. doi: 10.1371/journal.pntd.0006918 (PMC6211769; doi:10.1371/journal.pntd.0006918)
Supplement: S5 Table — (DOC) [file pntd.0006918.s008.doc]

**S5 Table. Logistic regression models for safety variables adjusted by age, sex, multiple lesions, and high-risk lesion.**

| **Adverse events** | **Praziquantel group (n=54)** | **Surgical group (n=42)** | **OR [95%CI]** |
| --- | --- | --- | --- |
| **Clinical events** | | | |
| Allergic reaction | 6 (11.1%) | 0 (0.0%) | 0.889[0.809, 0.977] |
| Headache | 10 (18.5%) | 8 (19.0%) | 1.031[0.348, 3.060] |
| Dizziness | 6 (11.1%) | 3 (7.1%) | 0.604[0.131, 2.773] |
| Sleepiness | 4 (7.4%) | 1 (2.4%) | 0.323[0.034, 3.102] |
| Abdominal pain | 4 (7.4%) | 0 (0.0%) | 0.379[0.037, 3.925] |
| Diarrhea | 3 (5.6%) | 1 (2.4%) | 0.550[0.049, 6.193] |
| **Laboratory events** | | | |
| ALT increase | 5 (9.3%) | 1 (2.4%) | 0.212[0.023, 1.945] |
| AST increase | 6 (11.1%) | 1 (2.4%) | 0.209[0.023, 1.924] |
| Creatinine increase | 3 (5.6%) | 2 (4.8%) | 0.799[0.120, 5.361] |
| BUN increase | 2 (3.7%) | 0 (0.0%) | 0.401[0.081, 1.991] |
| Proteinuria | 1 (1.9%) | 0 (0.0%) | 0.893[0.634, 1.257] |

Abbreviation: ALT=alanine aminotransferase; AST=aspartate aminotransferase; BUN= blood urea nitrogen.
