# Supplementary material for: Efficacy comparison between long-term high-dose praziquantel and surgical therapy for cerebral sparganosis: A multicenter retrospective cohort study
Source: PLoS Negl Trop Dis. 2018 Oct 22;12(10):e0006918. doi: 10.1371/journal.pntd.0006918 (PMC6211769; doi:10.1371/journal.pntd.0006918)
Supplement: S1 Checklist — (DOC) [file pntd.0006918.s009.doc]

STROBE Statement—Checklist of items that should be included in reports of ***cohort studies***

|  | Item No | Recommendation | Manuscript section and paragraph number | Text extracts |
| --- | --- | --- | --- | --- |
| Title and abstract | 1 | (*a*) Indicate the study’s design with a commonly used term in the title or the abstract | Title | Efficacy comparison between long-term high-dose praziquantel and surgical therapy for cerebral sparganosis: a multicenter retrospective cohort study |
|  |  | (*b*) Provide in the abstract an informative and balanced summary of what was done and what was found | Abstract, paragraph 1-3 | **Background:**  Sparganosis is a parasitic infection caused by the plerocercoid larvae of the *Spirometra mansoni* in East and Southeast Asia. The plerocercoid larvae sometimes invade the encephalon, resulting in severe cerebral sparganosis. Surgical removal of the larvae is considered a standard therapy for cerebral sparganosis. In contrast, the efficacy and safety of long-term, high-dose praziquantel treatment have not been determined in cerebral sparganosis.  **Methodology/Principal Findings:**  In this multicenter retrospective study, we assessed the records of 96 patients with cerebral sparganosis who consulted at 3 medical centers from 2013 to 2017. Forty-two patients underwent surgical lesion removal, and the other 54 patients received long-term, high-dose praziquantel (50 mg/kg/day for 10 days, repeated the treatment cycle at monthly intervals). The primary outcome was complete disappearance of the active lesions on cerebral magnetic resonance imaging. The secondary outcomes were the modified Rankin scale score at 90 days, incidence of seizure, eosinophil count, and serological *Spirometra. mansoni* antibody titer. The efficacy of praziquantel treatment was similar to that of surgical lesion removal for cerebral sparganosis with respect to both the primary outcome and secondary outcomes. Although binary logistic regression models also supported the primary outcome after adjustment for age, gender, lesion location, and loss to follow-up, some unavoidable confounders might have biased the statistical power. No significant clinical complications or laboratory side effects occurred in the praziquantel group with the exception of a relatively benign allergic reaction.  **Conclusions/Significance:**  In this small-sample, nonrandomized, retrospective exploratory study, some patients with cerebral sparganosis were responsive to long-term, high-dose praziquantel in comparison with surgical lesion removal. These findings increase the treatment flexibility for this serious infection. |
| Introduction | | |  |  |
| Background/rationale | 2 | Explain the scientific background and rationale for the investigation being reported | Introduction, paragraph 1-3 | Sparganosis is a type of parasitic zoonosis associated with infection by the larval cestode of *Spirometra* [1]. Most cases were reported in East and Southeast Asian countries, especially in China [2], South Korea [3], Japan [4], and Thailand [5]. Humans are infected with *Spirometra* by drinking water contaminated with procercoid-infected copepods, eating undercooked meat of snakes or frogs infected with *Spirometra*, or by using poultices of frog or snake flesh or skin on the open wounds [1, 6]. The plerocercoid larva usually affects the subcutaneous tissue or muscle in a human host [7]. However, it can also sometimes invade encephalon, resulting in severe cerebral sparganosis [8, 9], which can be manifested by headache, seizure, limb paralysis, aphasia, cognitive disorder, and other focal neurological deficits [10, 11]. Characteristic MRI findings such as aggregated ring-like enhancement, tunnel sign, and wandering lesions, are very useful to diagnose of cerebral sparganosis [12].  Although surgical removal of the parasite has been considered as a standard therapy [13], failure of this treatment in some cases was also reported [14, 15]. Surgical operation has been considered as the first line therapy for cerebral sparganosis just because treatments with anti-helminthics, including praziquantel, have been described as ineffective [16, 17]. Since praziquantel was first introduced as a broadspectrum antiparasitic drug in 1975, it has been proved as a successful treatment for the majority of human-infecting trematodes and cestodes including schistosomiasis, clonorchiasis, paragonimiasis, taeniasis, and cysticercosis [18]. However, the efficacy of praziquantel therapy for cerebral sparganosis has been controversial. Some patients who received conventional-dose praziquantel (25mg/kg/day for 3 days) alone even got worse [19].  In 2012, our group reported three cases with cerebral sparganosis showing good therapeutic outcome of high-dose of praziquantel (50 mg/kg/day for 10 days) [9]. In 2013, Roman and colleagues also found that high-dose praziquantel treatment (75 mg/kg/day for 7 days) was efficacious in an inoperable case of cerebral sparganosis [20]. Cerebral sparganosis is a severe and disable disease, but public health strategies have not made its prevention and treatment a priority. Therefore, it is worthy to investigate the efficacy and safety of high-dose praziquantel treatment for cerebral sparganosis, because the drug treatment may be convenient and cost-effective. In this study, we compared the clinical outcomes of patients treated with long-term high-dose praziquantel or surgical therapies through a multicenter retrospective cohort study. Our results showed that most patients with praziquantel therapy achieved favorable outcomes in the follow-up. |
| Objectives | 3 | State specific objectives, including any prespecified hypotheses | Introduction, paragraph 1 | The primary research objective was to evaluate the efficacy and safety of long-term, high-dose praziquantel treatment formula (50 mg/kg/day for 10 days) for cerebral sparganosis compared with surgical therapy through a multicenter retrospective study. |
| Methods | | |  |  |
| Study design | 4 | Present key elements of study design early in the paper | Methods, paragraph 1 | We conducted a retrospective study using routine program data. |
| Setting | 5 | Describe the setting, locations, and relevant dates, including periods of recruitment, exposure, follow-up, and data collection | Methods, paragraphs 1 | This was a multicenter retrospective study using data collected by physicians monitoring patients with cerebral sparganosis in three academic medical centers: the First Affiliated Hospital of Nanchang University, Peking University People’s Hospital, and Jiangxi Provincial Institution of Parasitic Diseases. The primary research objective was to evaluate the efficacy and safety of long-term, high-dose praziquantel treatment formula (50 mg/kg/day for 10 days) for cerebral sparganosis compared with surgical therapy. Because of the successful experience in our clinical centers before 2012 [9], we established a routine clinical procedure for cerebral sparganosis under supervision by Dr. Hong, Xie, and Wan in the three medical centers at the end of 2012. Therefore the clinical charts and imaging data were relatively complete in the retrospective review, but a quantitative assessment of the pretreatment disease severity was absent. All consecutive patients with cerebral sparganosis from January 2013 to December 2017 were retrospectively retrieved from the database. The patient list was compiled by searching the electronic medical records using the International Classification of Diseases Tenth Revision (ICD10) discharge code B70.151. |
| Participants | 6 | (*a*) Give the eligibility criteria, and the sources and methods of selection of participants. Describe methods of follow-up | Methods, paragraphs 1,2 | The inclusion criteria included: (1) patients showed cerebral symptoms associated with at least one structural lesion; (2) patients had definite evidence of sparganum infections that was proved by immunopositivity to *Spirometra mansoni* antibody in both serum and cerebrospinal fluid (CSF) tests and/or pathological evidences; (3) patients underwent follow-up cerebral MRI and serological immunological tests in our centers. The exclusion criteria included: (1) patients lost to follow-up; (2) patients with severe surgical contradictions associated with cardiopulmonary dysfunctions; (3) patients with contradictions for praziquantel treatment due to liver and renal dysfunctions; (4) operative patients initially received praziquantel; (5) patients with praziquantel formula treatment after surgical lesion removal.  The patients were classified into a surgical therapy group and long-term, high-dose praziquantel therapy group according to the following principles. All patients initially received a detailed clinical assessment, followed by medical education about cerebral sparganosis as a reference for choosing either praziquantel treatment or surgical treatment themselves. The physicians did not make a decision to treatment options for them, except patients with multiple lesions who would directly receive praziquantel treatment. The final treatment plan was chosen according to the willingness of the patients or their legal guardians. The disease severity and high-risky lesions located at some important brain regions might have been overemphasized in some communications, but the proportion could not be determined in the retrospective study. However, high-risky lesions were not considered an operative contraindication because our neurosurgeons had excellent technical skills and experience for the operation. The main reasons for treatment with long-term, high-dose praziquantel treatment were refusal to undergo surgery, lesions located at important functional areas of the brain, and initial tentative therapy (i.e., patients want to try two cycles of praziquantel treatment, if no positive therapeutic outcome showed up, they will transfer to surgical removal). |
|  |  | (*b*) For matched studies, give matching criteria and number of exposed and unexposed | _ | NA |
| Variables | 7 | Clearly define all outcomes, exposures, predictors, potential confounders, and effect modifiers. Give diagnostic criteria, if applicable | Methods, paragraphs 2,3,5 | The clinical manifestations of cerebral sparganosis were closely related to the site of lesions, and the clinical prognosis was significantly associated with the recovery of granulomatous lesions. The active ring-like or tunnel-like enhancements represented the direction of active inflammatory tunnels in different dimensions. Therefore, the primary efficacy endpoint was disappearance of the active lesion on contrast MRI. All patients received MRI scan before the treatment. The surgical patients had a follow-up MRI at 1 month after the surgery. The patients with praziquantel treatment had MRI scan at the beginning of the each treatment cycle. If the active lesions disappeared, the treatment with praziquantel would stop. If patients presented with neurological symptoms at any time after completion of the treatment regimen, cerebral MRI would be performed immediately. If the patients had new lesions after the active lesions disappeared, the case would be considered as a treatment failure.  The secondary efficacy endpoints included the clinical outcome assessed by the score on the modified Rankin scale (mRS) at 90 days after the end of the treatment, incidence of seizure, eosinophils counting, and serological titers of *Spirometra mansoni* antibody. The mRS is a 7-point scale ranging from 0 (no symptoms) to 6 (death). A score of 2 or less indicates functional independence. The seizure is defined as a clinical event occurred after the end of treatment regardless whether seizures existed before the treatment. Eosinophils counting were conducted at the end of the treatment, and the number of patients with eosinophils percentage higher than 5% was counted. Serological titers were only compared between the beginning and the end of treatment.  The clinical variables evaluated in this study were age, gender, epidemiological history, headache, seizure, hemiparesis, and aphasia. The epidemiological history judged by whether the patients had been infected with *Spirometra* *mansoni* by drinking water contaminated with procercoid-infected copepods, eating undercooked meat of snakes or frogs infected with *Spirometra mansoni*, or using poultices of frog or snake flesh or skin on open wounds. The laboratory variables were the cerebral MRI findings (aggregated ring-like enhancement, the tunnel sign, lesion migration, high-risky lesions, and multiple lesions), blood eosinophil percentage, and serological and CSF immunological results for a panel of parasite antibodies including spirometra mansoni, schistosoma japonicum, cysticercosis, paragonimiasis, clonorchiasis, toxoplasmosis, and echinococcosis. Aggregated ring-like enhancement refers to conglomerated ring-like enhancement, which is seen as bead shaped, usually 3 to 6 rings, on MRI (S1 Fig A). The tunnel sign is about 4 cm in length (usually 2-6 cm) and 0.8 cm in width (usually 0.5-1.5 cm), and exhibits marked enhancement on coronal and sagittal contrast MRI (S1 Fig B). Lesion migration indicates the presence of new and old lesions in different cerebral locations due to the migration of larva (S1 Fig C, D). Multiple lesions indicate that at least two active lesions located at different encephalic regions. A high-risky lesion is a lesion located at an important functional area of the brain, including the brain stem, thalamus, and precentral gyrus. The *Spirometra mansoni* IgG antibody titer was expressed as the optical density value on microplate enzyme-linked immunosorbent assay. The cut-off value of the optical density was 0.30 as determined by normal human serum in our laboratory.  The final treatment plan was chosen according to the willingness of the patients or their legal guardians. The disease severity and high-risky lesions located at some important brain regions might have been overemphasized in some communications, but the proportion could not be determined in the retrospective study. However, high-risky lesions were not considered an operative contraindication because our neurosurgeons had excellent technical skills and experience for the operation. The main reasons for treatment with long-term, high-dose praziquantel treatment were refusal to undergo surgery, lesions located at important functional areas of the brain, and initial tentative therapy (i.e., patients want to try two cycles of praziquantel treatment, if no positive therapeutic outcome showed up, they will transfer to surgical removal).  Among the 9 patients with loss of follow-up who were initially excluded in the study, 6 patients had not received any treatments after diagnosis, but other 3 patients initially received the praziquantel treatment for 2-3 cycles. Even if the 3 patients plus the 3 retreated patients were counted as negative primary outcome, the sub-analysis also showed no significant difference between praziquantel treatment and surgical removal (80.0% vs. 92.9%; p=0.091). Eleven out of 12 patient with multiple or risky lesions got the primary outcome in praziquantel treatment group, and all 4 patients with risky lesions got the primary outcome in surgical removal group. As for the possible bias confounder of lesion location, the sub-analysis revealed no significant difference between praziquantel treatment and surgical removal (91.7% [11/12] vs. 100% [4/4]; p=1.00). |
| Data sources/ measurement | 8 | For each variable of interest, give sources of data and details of methods of assessment (measurement). Describe comparability of assessment methods if there is more than one group | Methods, paragraphs 2, 6 | The clinical variables included age, gender, epidemiological history, headache, seizure, hemiparesis, and aphasia. Epidemiological history judged by whether patients were infected with *Spirometra* by drinking water contaminated with procercoid-infected copepods, eating undercooked meat of snakes or frogs infected with *Spirometra*, or by using poultices of frog or snake flesh or skin on the open wounds. The laboratory variables included cerebral MRI findings (aggregated ring-like enhancement, tunnel sign, lesion migration, risky lesion, and multiple lesions), blood eosinophile percentage, and serological and cerebrospinal fluid (CSF) immunological tests for a panel of parasite antibodies including *Spirometra mansoni*, *Schistosoma japonicum*, *Cysticercosis*, *Paragonimiasis*, *Clonorchiasis*, *Toxoplasmosis*, and *Echinococcosis*. The aggregated ring-like enhancement indicates a conglomerated ring-like enhancement, which is seen as bead shaped, usually 3 to 6 rings, on MR images (S1 Fig A). Tunnel sign is about 4 cm in length (usually 2-6 cm) and 0.8 cm in width (usually 0.5-1.5 cm), which shows marked enhancement on coronal and sagittal contrast MR images (S1 Fig B). Lesion migration means the presence of new and old lesions in different cerebral locations indicating the migration of larva (S1 Fig C, D). Multiple lesions mean that at least 2 active lesions located at different encephalic regions. Risky lesion indicates that the lesion locates at important functional areas of the brain including brain stem, thalamus, and precentral gyrus. *Spirometra mansoni* IgG antibody titer was expressed as the optical density value on microplate ELISA (enzyme-linked immunosorbent assay). Cut-off value of optical density was 0.30 determined by normal human serum in our labs.  The clinical symptoms included vital signs, headache, dizziness, sleepiness, abdominal pain, diarrhea, and any reported adverse events. The laboratory indexes included hematology, alanine aminotransferase, aspartate aminotransferase, blood creatinine, blood urine nitrogen, urine parameters, and electrocardiography. The allergy of praziquantel treatment for cerebral sparganosis usually presented with fever, chills, pruritus, and urticaria occurred in the first or second treatment cycles. The adverse events were defined as clinical symptoms with onset or worsening severity at or after the first dose of study drug through the safety follow-up visit (day 30). |
| Bias | 9 | Describe any efforts to address potential sources of bias | Methods, paragraph 1,2,9 | Since the program onset, clinical data were collected using standardized data collection tools and stored in electronic databases. The databases were updated on a daily basis by data managers.  To adjust for the confounders in this retrospective study, several binary logistic regression models were established in a sub-analysis to identify the outcomes. |
| Study size | 10 | Explain how the study size was arrived at | Methods, paragraph 1,2  Results, paragraph 1 | We conducted a retrospective cohort study using routine program data. In the main analysis, we included all infected patients diagnosed between January 2013 and December 2017 treated with praziquantel and surgical treatment. Patients that discontinued treatment, defaulted, were transferred-out or had a missing treatment outcome were excluded.  Fifty-one patients with long-term high-dose praziquantel treatment achieved complete lesion recovery at the end of treatment. Three patients still had active lesions after eight regimens of praziquantel treatment, and then accepted surgical removal. Among the 51 patients, 3 patients had recurrent lesions at the third, fourth and sixth month after the recovery of active lesions, respectively. Among patients with successful praziquantel treatment, 2 patients had a praziquantel regimen; 5 patients had 2 praziquantel regimens; 19 patients had 3 praziquantel regimens; 13 patients had 4 praziquantel regimens (Figure 2); 5 patients had 5 praziquantel regimens; 2 patients had 6 praziquantel regimens; 1 patient had 7 praziquantel regimens; 1 patient had 8 praziquantel regimens. The long-term high-dose praziquantel treatment demonstrated a similar efficacy to surgical removal for cerebral sparganosis at the primary efficacy endpoint (88.9% vs. 92.9%; p=0.727) (Table 2). Even if the cut-off of treatment regimens was set at 5, the primary efficacy endpoint still demonstrated no difference between praziquantel and surgical group (81.5% vs 92.9%; p=0.106). The number of patients who were classified as a score of 2 or less of mRS was similar between praziquantel and surgical group. No patients in both of groups were allocated in a mRS score of more than or equal to 5. The incidence of seizure after the end of treatment showed similar values between praziquantel and surgical group. The number of patient with eosinophils counting more than 5% and serological titer of Spirometra mansoni antibody were similar across the two treatment groups at the end of treatment. |
| Quantitative variables | 11 | Explain how quantitative variables were handled in the analyses. If applicable, describe which groupings were chosen and why | Methods, paragraph 1 | Continuous variables were categorized based on retrospective medical data. |
| Statistical methods | 12 | (*a*) Describe all statistical methods, including those used to control for confounding | Methods, paragraph 9 | Statistical methods  All statistical analyses were performed using the Statistical Package for the Social Sciences 17.0 software (SPSS, Inc., Chicago, IL, USA), and a p-value of <0.05 was considered statistically significant. Categorical variables were presented as count (percentage). Continuous variables were reported as mean ± standard deviation. The statistical significance of intergroup difference was assessed by pooled-variance and separate-variance Student’s t-test, The Chi-squared test, or Fisher's exact test as appropriate. To adjust for the confounders in this retrospective study, several binary logistic regression models were established in a sub-analysis to identify the outcomes. |
|  |  | (*b*) Describe any methods used to examine subgroups and interactions | ­ Methods, paragraphs 9  Result paragraph 4 and 5 | To adjust for the confounders in this retrospective study, several binary logistic regression models were established in a sub-analysis to identify the outcomes.  Several logistic regression models were configured to further evaluate the confounders that may have introduced bias into the retrospective study. After adjustment for age, gender, multiple lesions, and high-risky lesions, the long-term, high-dose praziquantel treatment showed efficacy similar to that of surgical lesion removal (Table 3 and S4 Table). Because the lesion locations was considered as be a possible major confounder causing selection bias, the patients with multiple lesions and high-risky lesions were excluded from a sub-analysis, which showed that the long-term, high-dose praziquantel treatment still had efficacy similar to that of surgical removal (Table 3). Of the nine patients who lost to follow-up and thus initially excluded from the study, six had not received any treatments after diagnosis, but the other three had initially received praziquantel for two to three cycles. Even when these three patients plus the three retreated patients were counted as a negative primary outcome in the praziquantel group, the sub-analysis adjusted for age, gender, multiple lesions, and high-risky lesions showed no significant difference between the praziquantel and surgical groups (Table 3). Considering that allergic reactions were only observed in the praziquantel group, the patients with allergic reactions were excluded from the sub-analysis, which showed that the praziquantel treatment still had efficacy similar to that of surgical removal (Table 3). |
|  |  | (*c*) Explain how missing data were addressed | Methods, paragraphs 1 | Patients that discontinued treatment, defaulted, were transferred-out or had a missing treatment outcome were excluded. |
|  |  | (*d*) If applicable, explain how loss to follow-up was addressed |  | Loss tofollow-up were excluded. |
|  |  | (*e*) Describe any sensitivity analyses |  | NA |
| Results | | |  |  |
| Participants | 13* | (a) Report numbers of individuals at each stage of study—eg numbers potentially eligible, examined for eligibility, confirmed eligible, included in the study, completing follow-up, and analysed | Results, paragraph 1, Fig 1 | The patient disposition and analysis were depicted in Figure 1. In total, 108 patients were screened by discharge code; 99 patients met the inclusion criteria; and nine patients were excluded due to lost of fellow-up. Initially, 42 patients accepted surgical removal for lesions, and 57 patients were administrated with long-term high-dose praziquantel. Three patients who initially accepted a tentative praziquantel treatment had removal operation, when no therapeutic effects were observed after two treatment regimens. Therefore, the number of patient was 42 in surgical group, and the number was 54 in praziquantel group. |
|  |  | (b) Give reasons for non-participation at each stage | - | NA |
|  |  | (c) Consider use of a flow diagram | Results, Fig 1 | Fig 1. Flowchart of study enrollment and rationale for exclusion. |
| Descriptive data | 14 | (a) Give characteristics of study participants (eg demographic, clinical, social) and information on exposures and potential confounders | Results, paragraph 1, Table 1 | Among these patients, there were no differences between patients enrolled in the surgical and praziquantel treatment arms in terms of demographics, epidemiological history, clinical manifestations, and relevant laboratory data (Table 1).  Table 1. Baseline characteristics between praziquantel and surgical treatment group. |
|  |  | (b) Indicate number of participants with missing data for each variable of interest | - | NA |
|  |  | (c) Summarise follow-up time (eg, average and total amount) | _ | NA |
| Outcome data | 15* | Report numbers of outcome events or summary measures over time | Results, paragraph 1,2, 4 Table 2 | All 42 patients in the surgery group initially underwent surgical lesion removal (24 by craniotomy and 18 by CT-guided stereotactic aspiration) and achieved complete lesion recovery 1 month after surgery. However, three patients developed new lesions during the postoperative follow-up: one lesion appeared in the second month after stereotactic aspiration, one reoccurred in the third month after craniotomy, and one reoccurred in the fifth month after stereotactic aspiration. The treatment effects between craniotomy and CT-guided stereotactic aspiration were not significantly different (95.8% vs 88.9%; absolute difference, 6.9%; 95% confidence interval [-23.9%, 36.9%]; p=0.567; Fisher's exact test).  Fifty-one patients who underwent long-term, high-dose praziquantel treatment achieved complete lesion recovery at the end of treatment. Three patients still had active lesions after eight cycles of praziquantel treatment, and then underwent surgical removal. Among the 51 patients, 3 had recurrent lesions at the third, fourth, and sixth month after recovery of the active lesions, respectively. Among the patients who achieved successful praziquantel treatment, 2 underwent 1 treatment cycle; 5 underwent 2 treatment cycles; 19 underwent 3 treatment cycles; 13 underwent 4 treatment cycles (Fig 2); and 9 required 5 to 8 treatment cycles (S2 Table and S2 Fig). The long-term, high-dose praziquantel treatment for cerebral sparganosis showed efficacy similar to that of surgical removal with respect to the primary efficacy endpoint (88.9% vs. 92.9%; p=0.727) (Table 2). Even when the cut-off of the number of treatment cycles was set at five, the primary efficacy endpoint still showed no significant difference between the praziquantel and surgical groups (81.5% vs 92.9%; p = 0.106). No patients in either group had a mRS score of more than or equal to 5. The number of patients with a mRS score of 2 or less was similar between the praziquantel and surgical groups. The incidence of seizures after the end of treatment was similar between the praziquantel and surgical groups. The numbers of patients with an eosinophil count of more than 5% and serological *Spirometra mansoni* antibody titer were similar between the two groups at the end of treatment (Table 2).  Six patients developed allergic reactions in the praziquantel group, but no patients developed allergic reactions in the surgical group. Although a difference was observed in the numbers of patients with allergic reactions between the two groups (Table 4), the clinical course of the allergic reactions was relatively benign and rapidly resolved after administration of 5mg/day of dexamethasone. Headache, dizziness, abdominal pain, diarrhea, and sleepiness were also reported by the patients. The incidence of these symptoms was similar between the praziquantel and surgical groups (Table 4). No differences in vital signs or electrocardiography findings were identified between the two groups. Increases in the aspartate aminotransferase (AST) and alanine aminotransferase (ALT) indicated a possibility of liver dysfunction, which occurred in a small proportion of the patients in the first or second treatment cycle. The dysfunction was characterized by two- to four- fold elevations of the AST/ALT without jaundice and was resolved by symptomatic treatment. Overall, no clinically meaningful differences in these laboratory abnormalities were observed between the two groups (Table 4). No patients withdrew from the study because of adverse events. Binary logistic regression models adjusted for age, gender, multiple lesions, and high-risky lesion further showed that these safety variables were not different between the two groups (S5 Table). |
| Main results | 16 | (*a*) Give unadjusted estimates and, if applicable, confounder-adjusted estimates and their precision (eg, 95% confidence interval). Make clear which confounders were adjusted for and why they were included | Result  Paragraph 3, 4 | Several logistic regression models were configured to further evaluate the confounders that may have introduced bias into the retrospective study. After adjustment for age, gender, multiple lesions, and high-risky lesions, the long-term, high-dose praziquantel treatment showed efficacy similar to that of surgical lesion removal (Table 3 and S4 Table). Because the lesion locations was considered as be a possible major confounder causing selection bias, the patients with multiple lesions and high-risky lesions were excluded from a sub-analysis, which showed that the long-term, high-dose praziquantel treatment still had efficacy similar to that of surgical removal (Table 3). Of the nine patients who lost to follow-up and thus initially excluded from the study, six had not received any treatments after diagnosis, but the other three had initially received praziquantel for two to three cycles. Even when these three patients plus the three retreated patients were counted as a negative primary outcome in the praziquantel group, the sub-analysis adjusted for age, gender, multiple lesions, and high-risky lesions showed no significant difference between the praziquantel and surgical groups (Table 3). Considering that allergic reactions were only observed in the praziquantel group, the patients with allergic reactions were excluded from the sub-analysis, which showed that the praziquantel treatment still had efficacy similar to that of surgical removal (Table 3). |
|  |  | (*b*) Report category boundaries when continuous variables were categorized | Results,  Table 2 | Table 2. Results of the primary and secondary efficacy outcomes between praziquantel and surgical treatment group. |
|  |  | (*c*) If relevant, consider translating estimates of relative risk into absolute risk for a meaningful time period | _ | NA |
| Other analyses | 17 | Report other analyses done—eg analyses of subgroups and interactions, and sensitivity analyses | - | NA |
| Discussion | | |  |  |
| Key results | 18 | Summarise key results with reference to study objectives | Discussion, paragraph 2. | In this study, long-term high-dose of praziquantel showed a similar efficacy to surgical removal in cerebral sparganosis for both the primary efficacy endpoint (active lesions in MRI) and secondary efficacy endpoint (mRS at 90 days, seizure, eosinophils counting and serological titer). Efficacy demonstrated most patients with cerebral sparganosis were sensitive to praziquantel with 50 mg/kg/day in three divided doses for 10 days at the third or fourth treatment regimen. The demographic characteristics, epidemiological history, clinical features, radiological changes, and laboratory tests between surgical group and praziquantel group showed no significant differences. Failure rates for praziquantel and surgical removal were balanced, but the reasons for failure were not similar across the treatment groups. |
| Limitations | 19 | Discuss limitations of the study, taking into account sources of potential bias or imprecision. Discuss both direction and magnitude of any potential bias | Discussion, paragraph 2, 5. | Quantitative assessments of disease severity before therapy were unavailable in this retrospective study. Naturally, it was possible that patients with milder severity whose clinical course showed little progression and who may not need invasive surgery might select praziquantel treatment; additionally, patients with complicated clinical course or multiple or high-risky lesions might not select surgical removal. As a result, although there were no significant differences in the available baseline data between the praziquantel and surgical groups, some confounders could have biased the primary outcome. To compensate for this bias, several logistic regression models were established to adjust for age, gender, lesion location, and loss to follow-up. These regression models showed that praziquantel still had efficacy similar to that of surgery, but some of these results actually showed borderline significance and indicated a tendency toward a surgical benefit.  This study had some limitations that need to be explicitly acknowledged. First, it was a small-sample, nonrandomized retrospective study; thus the power of significance was low. Cases of cerebral sparganosis are rare, so it is necessary to include data from more medical centers. Second, the fact that the patients made the treatment decision themselves undermined the reliability of the results. Confounders such as the disease severity, multiple lesions, high-risky lesions, and doctor-patient communication skills might have produced some bias with respect to the treatment decision. Third, the clinical data were quite heterogeneous because the treatment with praziquantel was different in each patient based on the lesion site and response. It was impracticable to assess the outcome only once and at a fixed time to avoid biasing the results because of ethical considerations and the pharmacological properties of praziquantel. Overall, the outcomes of this study should be cautiously interpreted; however, they seemed to indicate that long-term, high-dose praziquantel had efficacy similar to that of surgical removal, and the logistic regression models supported these results. Indeed, several baseline outcomes and safety indexes were borderline significant, which indicated a benefit tendency of surgical removal for larva lesion. |
| Interpretation | 20 | Give a cautious overall interpretation of results considering objectives, limitations, multiplicity of analyses, results from similar studies, and other relevant evidence | Discussion, paragraphs 3, 5 | Overall, the outcomes of this study should be cautiously interpreted; however, they seemed to indicate that long |
| Generalisability | 21 | Discuss the generalisability (external validity) of the study results | Discussion, paragraph 5. | In this study, long-term, high-dose praziquantel resolved the cerebral lesions in 88.9% of patients with cerebral sparganosis. Most patients with cerebral sparganosis achieved effective treatment by praziquantel at 50 mg/kg/day for 10 days at the third or fourth treatment cycle. The absolute efficacy rate of praziquantel was relatively lower than that of surgical removal, but there were no significant differences in the primary efficacy endpoint (active lesions on MRI) and secondary efficacy endpoints (mRS score at 90 days, seizure, eosinophil count, and serological titer) between the praziquantel and surgical groups. Quantitative assessments of disease severity before therapy were unavailable in this retrospective study. Naturally, it was possible that patients with milder severity whose clinical course showed little progression and who may not need invasive surgery might select praziquantel treatment; additionally, patients with complicated clinical course or multiple or high-risky lesions might not select surgical removal. As a result, although there were no significant differences in the available baseline data between the praziquantel and surgical groups, some confounders could have biased the primary outcome. To compensate for this bias, several logistic regression models were established to adjust for age, gender, lesion location, and loss to follow-up. These regression models showed that praziquantel still had efficacy similar to that of surgery, but some of these results actually showed borderline significance and indicated a tendency toward a surgical benefit.  Overall, the outcomes of this study should be cautiously interpreted; however, they seemed to indicate that long-term, high-dose praziquantel had efficacy similar to that of surgical removal, and the logistic regression models supported these results. Indeed, several baseline outcomes and safety indexes were borderline significant, which indicated a benefit tendency of surgical removal for larva lesion. However, this exploratory study conducted by real-world practitioners with much experience add treatment flexibility for this serious infection, and provide a basis to promote a large-sample randomized, prospective study of long-term, high-dose praziquantel treatment for cerebral sparganosis in the future. |
| Other information | | |  |  |
| Funding | 22 | Give the source of funding and the role of the funders for the present study and, if applicable, for the original study on which the present article is based | - | The funders had no role in study design, data collection and analysis, decision to publish, or preparation of the manuscript. |

Note: An Explanation and Elaboration article discusses each checklist item and gives methodological background and published examples of transparent reporting. The STROBE checklist is best used in conjunction with this article (freely available on the Web sites of PLoS Medicine at http://www.plosmedicine.org/, Annals of Internal Medicine at http://www.annals.org/, and Epidemiology at http://www.epidem.com/). Information on the STROBE Initiative is available at http://www.strobe-statement.org.
